# Nanoparticles in Targeted Alpha Therapy

**DOI:** 10.3390/nano10071366

**Published:** 2020-07-13

**Authors:** Agnieszka Majkowska-Pilip, Weronika Gawęda, Kinga Żelechowska-Matysiak, Kamil Wawrowicz, Aleksander Bilewicz

**Affiliations:** Centre of Radiochemistry and Nuclear Chemistry, Institute of Nuclear Chemistry and Technology, Dorodna 16, 03-195 Warsaw, Poland; w.maliszewska@ichtj.waw.pl (W.G.); k.zelechowska@ichtj.waw.pl (K.Ż.-M.); k.wawrowicz@ichtj.waw.pl (K.W.); a.bilewicz@ichtj.waw.pl (A.B.)

**Keywords:** α-emitters, radiopharmaceuticals, nanoparticles, nanomedicine, targeted therapy, tumour

## Abstract

Recent advances in the field of nanotechnology application in nuclear medicine offer the promise of better therapeutic options. In recent years, increasing efforts have been made on developing nanoconstructs that can be used as carriers for immobilising alpha (α)-emitters in targeted drug delivery. In this publication, we provide a comprehensive overview of available information on functional nanomaterials for targeted alpha therapy. The first section describes why nanoconstructs are used for the synthesis of α-emitting radiopharmaceuticals. Next, we present the synthesis and summarise the recent studies demonstrating therapeutic applications of α-emitting labelled radiobioconjugates in targeted therapy. Finally, future prospects and the emerging possibility of therapeutic application of radiolabelled nanomaterials are discussed.

## 1. Introduction

Radiation therapy is an important component of cancer treatment with approximately 50% of all cancer patients receiving radiation therapy during their course of illness [1]. The key objective in cancer radiotherapy is to achieve a high therapeutic efficacy by maximising damage to the tumour while minimising damage to surrounding healthy tissue. In contrast to radiation therapy methods that use an external ion beam source, internal radiotherapy is performed by the direct administration of radionuclides conjugated to a targeting vector. This can be achieved by coupling suitable radionuclides to antibodies, antibody fragments, nanobodies or small peptides that bind cell surface receptors or other proteins specifically overexpressed by cancer cells.

At the beginning, beta (β)-emitting radionuclides were widely used in cancer therapy. There are two registered therapeutic antibodies: tositumomab labelled with ^131^I (BEXXAR^TM^) that is used to treat follicular lymphoma, and ibritumomab labelled with ^90^Y (Zevalin^®^) is used to treat B cell non-Hodgkin’s lymphoma [2]. Furthermore, ^90^Y and ^177^Lu-labelled peptides, like somatostatin and bombesin analogues, showed promising results in current clinical trials [3]. In February 2018, the U.S. Food and Drug Administration (FDA) approved DOTATATE labelled with ^177^Lu (Lutathera^®^) for the treatment of certain neuroendocrine tumours [4]. One of the limitations of β^-^ emitting radionuclide therapies is their inability to treat small clusters of cancer cells like micrometastatic cancers or single leukaemia cells. This is because long ranges of the β^-^ particles cause death in normal healthy cells [5]. For example, *β^-^*-particles from ^90^Y (E_β(max)_) = 2.3 MeV deposit their energy over a range of 12 mm. The effective tissue range of *β^-^*-particles is not optimal for the treatment of tumours as small clusters of cells or of single cells and micrometastases, because much of the decay energy is deposited outside the boundary of the tumour. On the other hand, targeted radiotherapy using *α*-particles is a promising alternative to that based on *β^-^*-particles, because *α*-particles deposit whole of their energy within a few cell diameters (50–100 µm) [6]. The *α*-particle, a ^4^He nucleus, is relatively heavier than other subatomic particles emitted from decaying radionuclides and has a much shorter range in tissues. Compared with β^-^-particles, α-particles provide a very high relative biological effectiveness, killing more cells with less radioactivity. Accordingly, α-particles have roughly 500 times higher cytotoxic potency than *β*^-^-particles [7]. Just 15 α tracks through the nucleus of a cell are sufficient to cause apoptosis due to their high energy deposited per unit distance travelled (approximately 80 keV/mm) [8].

Recently, it has been found that in some cancers, such as in the case with leukaemia, breast and brain cancer, small subpopulations of tumour cells are able to self-renew and reconstitute the heterogeneous tumour cell population [9,10]. These stem-like cancer cells are also thought be involved in the widespread metastatic dissemination of cancer [11]. These findings suggest that failure in cancer treatment may be associated with the failure to eradicate cancer stem cells [12]. Cancer stem cells are usually resistant to chemotherapy, as well as external and internal radiotherapy including β^-^-emitters [13]. For this reason, effective targeted therapies for the complete eradication of these cells are urgently needed. Given the properties outlined above, tumour stem cells are ideal targets for targeted α particle therapy [14]. For example, Substance P labelled with α-emitter ^225^Ac is highly cytotoxic to chemo- and radioresistant glioblastoma multiforme (GBM) stem cells [15].

As with any medical methods, there are some limitations using α-particle therapy. First, is the availability. There are more than 100 radionuclides that decay by α-emission, but the majority of these radionuclides have half-lives either too short or too long for any therapeutic use, their production is not economically viable or chemical properties do not allow their use in medicine. Currently, the main α-emitters used for targeted therapy are ^223,224^Ra, ^211^At, ^225^Ac, ^212^Pb, ^227,226^Th and ^212,213^Bi. Table 1 summarises the main nuclear properties of medically used α-emitters.

However, these radionuclides have shortcomings. In the case of ^212^Bi, ^213^Bi and ^226^Th, the short half-life often limits the application of these nuclides to situations where tumour cells are rapidly accessible to the targeting agent. Moreover, ^212^Bi shows high-energy α-emission with 32% abundance that is absent in ^213^Bi. Therefore, the latter is generally considered to be a more attractive candidate for α-radiotherapy. In the case of ^225^Ac, ^223^Ra and ^227^Th, recoiling of α-emitting decay products from radiobioconjugates can accumulate in critical organs. ^225^Ac decays directly to ^221^Fr (alkali metal) that has a half-life of 4.9 min and then escaping from the ^225^Ac-radiobioconjugate. A similar situation appears in the case of ^227^Th and ^223^Ra where the decay product, the gaseous ^219^Rn, easily liberates itself from ^227^Th and ^223^Ra radiobioconjugates. It is worth mentioning that this problem is reduced in the case of ^223^Ra series because 75% of its total alphas are delivered within a few seconds (t_1/2_ = 4 s) after the ^223^Ra decay [16].

Additionally, the lack of appropriate bifunctional ligands is the reason why radium radionuclides did not find application in a receptor targeted therapy. The Ra^2+^ cation, like other cations of group 2, forms weak complexes. Therefore, labelling the biomolecules with ^223^Ra is a very difficult task. Until now, only ^223^Ra in the simple form of RaCl_2_, with its natural affinity to build into bones, finds application in the treatment of bone metastases from breast and prostate cancer [17]. The second alpha emitter whose binding to the biomolecule is a challenging is ^211^At.

In the last two years, a number of review articles on targeted α therapy [18,19,20,21] as well as several reviews concerned radioactive nanoparticles and their use in diagnostics and therapy have been published [22,23,24]. These reviews contain also some information on nanoparticles labelled with α emitters; however, this issue is briefly described. Below we summarise existing works on α emitter radiolabelled functional nanomaterials and provide illustrative examples of their application in nuclear nanomedicine. The first section focuses on the problem of the reasons for the use of nanostructures in targeted α therapy. Next various nanomaterials investigated thus far and biomedical results of these studies are briefly described. Finally, future perspectives and the emerging potential for medical application of alpha emitter labelled nanomaterials are discussed.

## 2. Why Nanoconstructs Are Used for α Radionuclide Therapy?

There are three important reasons why nanoparticles are used in α radionuclide therapy. The first two are related to the difficulty of attachment of the radionuclides to the targeting vectors.

- Release of daughters from the radioisotopes, that are the mother radionuclides for the decay chains (^225^Ac, ^223^Ra, ^227^Th and ^212^Pb)
- Lack of appropriate bifunctional ligands for effective binding of α–emitters to targeted molecules (^211^At and ^223^Ra)
- Application of α-emitter-labelled nanoparticles in new therapeutic approach–targeted nanobrachytherapy.

These reasons are described in more detail below.

### 2.1. Controlling the Recoil of the Daughter Radionuclides

**Figure 1:**
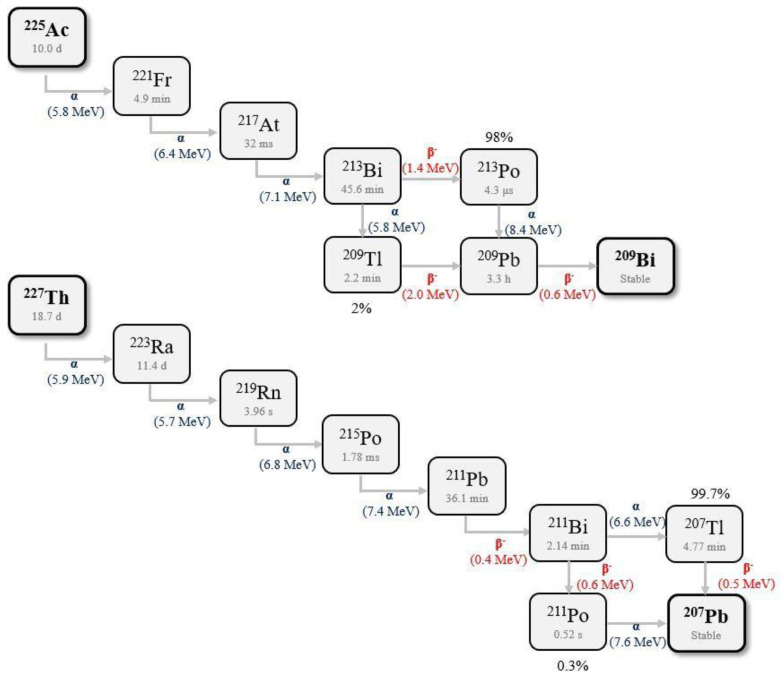
Decay chains of ^225^Ac and ^227^Th-^223^Ra radionuclides.

Radionuclides ^225^Ac, ^227^Th and ^223^Ra are good candidates for α therapy because they have a relatively long half-life and high cumulative decay energy (>28 MeV). However, all of these α-emitters have four or five unstable daughter nuclides that often emit α-particles as well. Figure 1 presents decay chains for ^225^Ac and ^227^Th-^223^Ra radionuclides. Important limitation for the application of these α-emitters in radionuclide therapy is the escape of daughter radionuclides from radiopharmaceuticals.

From the simple formula of the conservation of angular momentum, the decay energy is divided between the nucleus and the α particle:

(1) $$E_{r}=Q\frac{m_{\alpha }}{M_{r}}$$

where *E*_r_ is the recoil energy, *m*_α_ the rest mass of α particle, *M*_r_ mass of the recoil nucleus and *Q* is the decay energy. Because the mass of an α-particle is 4 amu (atomic mass units) and the mass of recoiled nucleus is ~210–220 amu, the energy distribution between the α-particle and the recoiling atom is typically as 1 to 50 [25].

As the energy of α decay is usually between 4 and 8 MeV, the daughter nuclide typically receives ~0.1 MeV of recoil energy. This is ~1000 times higher than the chemical binding energy, meaning the daughter radionuclide cannot be held by a chemical bond. Therefore, the sequestration of daughter radionuclides in chelating ligands, such as cyclic or linear polyamino carboxylate chelators like 1,4,7,10-tetraazacyclododecane-1,4,7,10-tetraacetic acid (DOTA) or diethylenetriaminepentaacetic acid (DTPA), is not possible. In this case, all daughter nuclides produced from α-emitting radionuclides are released from their ligand in vivo, limiting the dose that can be delivered to the target cells. After dissociating from the radiobioconjugate, free daughter radionuclides may cause harm to healthy tissue and initiate secondary tumourigenesis. This is particularly true when the daughter nuclides themselves are α-emitters. The transfer of the daughter radionuclide depends on its half-life, diffusion and affinity for certain organs. ^213^Bi from ^225^Ac decay migrates to the kidney causing renal toxicity. This renal toxicity can be partially moderated through the use of scavengers or addition of non-radioactive Bi^3+^. However, kidney toxicity remains a main limitation to application of ^225^Ac in radiotherapy [26,27]. Schwartz et al. [28] evaluated the contribution of nonequilibrium ^213^Bi to kidney dose in mice via γ-ray spectroscopy. The average absorbed dose into the kidneys was 0.77 Gy·kBq^−1^, where 60% was attributed to nonequilibrium ^213^Bi excess. There is less of a problem is with the ^223^Ra series because 75% of its total alphas are delivered within a few seconds (t_1/2_ = 4 s) after the ^223^Ra decay. This problem is more pronounced with the ^225^Ac series, because the ^225^Ac decays directly to ^221^Fr that has a t_1/2_ = 4.9 min. Additionally, as an alkali metal cation, it can be transported over a relatively long distance [16].

As discussed in the recent review by de Kruijff et al. [29], there are three different approaches to deal with this recoil problem: cell internalisation, local administration or encapsulation of α-emitters in nanocarriers.

Cell internalisation approach assumes the accumulation of radiopharmaceuticals inside cancer cells and keeps all daughter nuclides in the target cells. The remaining not adsorbed part of the radioconjugate is excreted fast from the body. The volume of the cell is usually large enough to keep inside most recoiling daughter radionuclides. This can be achieved only when the blood circulation time of radiobioconjugates is short and radiopharmaceutical rapidly accumulates inside the cancer cells. This strategy was applied to α-emitter-labelled internalised peptides like vascular tumour-homing peptide F3 [30], octreotide [31] and small fragments of monoclonal antibodies such as nanobodies [32]. However, this is particularly problematic in the case of ^225^Ac-labelled radiopharmaceuticals where ^221^Fr (t_1/2_ = 4.9 min) is first decay product. Francium as potassium analogue excreted from the cell by the Na^+^/K^+^ pump with subsequent decay (generation of the rest of the 3 α-emissions) happening outside the target cells [23].

The next approach is injecting the α-emitting radionuclides locoregionally in or near the tumour tissue, or in the cavity after tumour resection. The radiobioconjugate must be applied in a region with no or slow exchange with the surrounding tissue in order to ensure that no daughter radionuclides may infiltrate blood circulation [23]. Such strategy has been tested in Phase I clinical studies with ^213^Bi-DOTA-substance P locally injected in gliomas by Cordier et al. [33] and Królicki et al. [34]. More recently, a pilot study on the locoregional treatment of bladder cancer (carcinoma in situ) using the ^213^Bi-labelled anti-EGFR monoclonal antibody cetuximab was conducted in collaboration of Joint Research Center Karlsruhe and Technical University Munich, Germany [35]. The therapy was found to be safe and without any side effects as no activity of ^213^Bi was detected outside the bladder. Królicki et al. initiated a dose escalation study investigating the intratumoural/intercavitary injection of ^225^Ac-DOTAGA-[Thi^8^, Met(O_2_)^11^]-substance P [36]. The patients were treated with activities ranging from 10 to 42 MBq ^225^Ac-DOTAGA-[Thi^8^, Met(O_2_)_11_]-substance P. The treatment was well tolerated and the analysis of therapeutic efficacy and patient recruitment is ongoing. Preliminary results indicate negligible diffusion of ^213^Bi from the injection site.

The third option for preventing escape of daughter radionuclides from the target site is encapsulate the mother radionuclide in a nanoparticle that is big enough to physically sequester all recoils in the decay chain in its structure. The major advantage of incorporating ^225^Ac, ^223^Ra and ^227^Th within the nanoparticles is that the daughter radionuclides are sequestered at the site of targeting, preventing the nonspecific radiotoxicity. The size, shape and type of material needed to fully encapsulate ^225^Ac decay products have been comprehensively analysed by Holzwarth et al. [37]. They calculated the probability of retention ^225^Ac decay products in three-dimensional space after α disintegration of completely random orientations. They found that 12 nm gold layer or 39 nm graphite layer may prevent the release of ^221^Fr, the first decay product of ^225^Ac. To sequester all radionuclides up to the third daughter, a layer of 35 nm gold or 143 nm of graphite is necessary. However, it should be noted that for these calculations the authors assumed that the mother radionuclides of the decay chains must be localised at the centre of the spherical nanoparticles. We describe this problem in more detail presenting experimental results below.

### 2.2. Nanoparticles as Agent for Binding of Alpha with Biomolecules

Another important limitation that affects the use of targeted alpha therapy is the availability and price of the radionuclides. ^211^At and ^223^Ra radionuclides are readily available. Unfortunately, the difficulty of stable attachment of these radionuclides to the carrier biomolecule substantially limits their use in targeted α therapy. ^223^Ra decays to stable lead and bismuth through a cascade of short-lived α- and β^-^-particle emitters, releasing a total energy of ~28 MeV (Figure 1). Only ^223^Ra in the simple ionic form of RaCl_2_ has application in radiotherapy for the treatment of bone metastasis. Ra^2+^ cations, like other cations of the 2 group Mendeleev Table, form very weak complexes. Therefore, labelling biomolecules with ^223^Ra is a very difficult task. The stability constant of the Ra-DOTA complex is unknown, but using log K value for Ba^2+^ (12.6) [38] and difference between K values of Ba-EDTA = 9.88 ± 0.11 and Ra-EDTA = 9.11 ± 0.09 [39] can be estimated as 12 and is 10 orders of magnitude lower than for the Ac-DOTA complex.

Previous studies on binding of ^223^Ra to biomolecules by complexation of Ra^2+^ by tetraazacarboxylic acids, cryptands and calixarenes were unsuccessfully. Henriksen et al. [40] compared the most suitable ligands for complexation of ^223^Ra, cyclic compounds 1,4,7,10-tetraazacyclododecane- 1,4,7,10 tetraacetic acid (DOTA), 4,7,13,16,21,24-hexaoxa-1,10-diazabicyclo8.8.8-hexacosane (Kryptofix2.2.2), 5,11,17,23-tetra-tert-butyl-25,26,27,28-tetrakis(carboxymethoxy), calix[4] arene-tetraacetic acid (calix[4]-tetraacetic acid) and the open-chain chelator diethylene triamine-N;N′;N″-pentaacetic acid (DTPA). Based on the relative extraction constants, calix[4]-tetraacetic is the most promising of the compounds tested. However, rapid dissociation indicates that the calix[4]-derivative is not suitable for in vivo application. Recently, Gott et al. [41] employed an innovative approach using polyoxopalladates to stably incorporate radium radionuclides as central cations. During the synthesis, the polyanion is formed with Ra^2+^ as central and counter ions. Unfortunately, after chromatographic purification, a portion of the unincorporated Ra^2+^ remains on the polyanion surface. This hinders the use of this complex. Therefore, the use of nanoparticles to joint ^223^Ra with biological vectors can be a best way for the application of ^223^Ra in targeted α therapy.

Cyclotron obtained ^211^At is also a promising candidate for targeted α-radiotherapy because is 7.2 h half-life assures sufficient time for its transportation, synthetic chemistry, multistep labelling, quality control and clinical application without problems caused by the daughter emitting α-particles. Another merit of this radionuclide is the simplicity of its production in cyclotrons in ^209^Bi(α,2n)^211^At nuclear reaction, followed by simple dry distillation and isolation from irradiated bismuth target. A variety of ^211^At labelled biomolecules have been examined in preclinical models; two have reached the stage of initial clinical trials [42,43]. Unfortunately, initial results were not positive due to instability of the astatine–biomolecule bond in biological fluids. Astatine is a member of the halogen group and shows some metallic character in the + 1 oxidation state [44]. The energy carbon–halogen bond for astatine is significantly lower than for iodine, which excludes the use of elaborated iodination methods for labelling biomolecules with ^211^At [45]. More stable astatinated proteins have been prepared by acylation of astatobenzoic acid derivatives prepared from trialkylstannyl precursors [46]. Unfortunately, biomolecules labelled by this method is not always stable with respect to in vivo deastatination [47]. Because of these difficulties, non-traditional solutions labelling carboranes [48], calixarenes [49], astatine hypervalent compounds [50] and ^211^At-Rh(III) [51] complexes have been reported with little success. Therefore, we believe that the use of nanostructures can solve this problem. The attempts of such an approach are discussed below in the discussion of individual radionuclides.

### 2.3. Application of α-Emitter Labelled Nanoparticles in New Therapeutic Approach—Targeted Nanobrachytherapy

Systemic radiotherapy involves the delivery of a soluble substance into the body, usually by injecting a radiopharmaceutical, which accumulates in the pathologically changed tissues. Another type of internal radiation therapy called brachytherapy involves placing closed radiation sources within or near the tumour using minimally invasive procedures. However, they require a complicated implantation technique under general anaesthesia. Furthermore, seed migration may also occur after implantation, and seed removal is required [52].

Recently, Reilly et al. proposed a novel targeted nanobrachytherapy approach for the treatment of locally-advanced breast cancer. This strategy uses intratumoural injection of 30 nm diameter gold nanoparticles modified with polyethylene glycol (PEG) chains with DOTA ligand that complex the therapeutic radionuclide ^177^Lu and linked to panitumumab that bind the AuNP to epidermal growth factor receptor (EGFR)-positive tumour cells or linked to Trastuzumab that bind to HER2 receptors [53,54]. Their studies on organ distribution shows that Au-Trastuzumab delivered intratumourally are retained (~30% ID/g) with minimal uptake by the liver and spleen [55]. This approach has also been proposed for ^111^In radiobioconjugates for application in Auger electron therapy [56].

There are several advantages of this type of injectable approach compared with conventional brachytherapy. Administration is easier and less invasive. Radiolabelled nanoparticles are in a microscopically dispersed in liquid form and can be injected intra- or peritumourally by syringe and needle. This strategy is less invasive than inserting solid seeds into the body. Additionally, their nanometer size permits local diffusion from the injection site, thus further homogenising the radiation dose deposition in the tumour [52]. Multiple small injections can achieve a more homogeneous distribution of the gold nanoseeds. Recently, a number of studies have extended this method to α-emitters such as ^211^At and ^225^Ac. This will be discussed in Section 3.

## 3. Nanoconstructs Labelled with α-Emitters

In Section 2, we presented the reasons why various nanoconstructs were used to immobilise α-emitters. Table 2 shows the data for labelled nanomaterials, summarised from the available literature.

### 3.1. Radium-223

**Figure 2:**
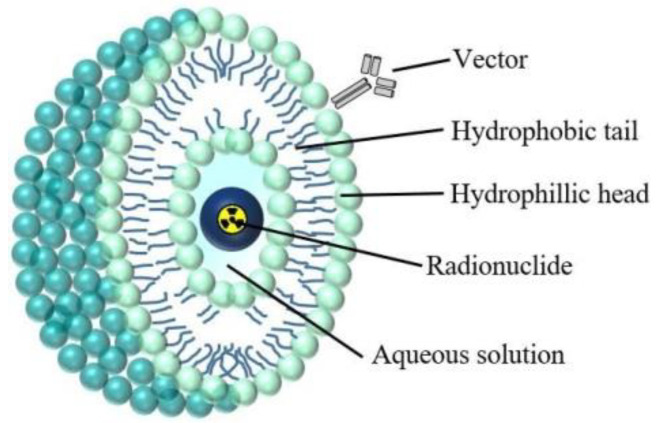
Structure of the liposome.

^223^Ra in the simple form of radium dichloride ([^223^Ra]RaCl_2_) is the first α-particle emitting therapeutic agent approved by the FDA for bone metastatic castration-resistant cancers [102]. However, as mentioned before, Ra^2+^ does not form stable complexes with conventional bifunctional chelators, similar to most alkali earth metals. To extend the use of ^223^Ra to other than bone metastases applications, several nanomaterials have been tested for stable incorporation of radium and linking to the targeting vector. The first studies used liposomes. As presented on Figure 2 liposomes are spherical vesicles having at least one lipid bilayer.

Liposomes have an aqueous solution core surrounded by a hydrophobic membrane. Hydrophilic solutes that are dissolved in the core cannot readily pass through the bilayer membrane [103]. Liposome drug delivery systems have produced substantial results in cancer therapy with various products reaching the marketing phase. In 1995, the first FDA-approved nanodrug was Doxil^®^, a pegylated liposome containing doxorubicin. It was reported that Doxil^®^ liposomes preferentially accumulate in mouse model tumours [104] as well as in patients with primary and metastatic disease [105]. In the first studies on immobilisation of ^223^Ra in liposomes, authors demonstrated that liposome-encapsulated radium could be prepared from preformed liposomes by ionophore-mediated loading [16]. ^223^Ra was incorporated with a good loading yield and was stably retained for several days when incubated at 37 °C in serum. In the next studies, Larsen et al. [57] used pegylated liposomal doxorubicin Caelyx^®^/Doxil^®^ drug to synthesise radiobioconjugates containing ^223^Ra that were functionalised by two vectors: folic acid and fragment of monoclonal antibody F(ab’)2(IgG1). These biodistribution studies showed the blood clearance of injected liposomal radium was much slower than the free ^223^Ra, as expected for pegylated liposomes. Among soft-tissue organs, the highest uptake was observed in the liver and spleen. Bone uptake also increased with time, most likely because ^223^Ra liposomes are metabolised by macrophages in the reticuloendothelial system. This process generates free cationic ^223^Ra, which is eliminated either by intestinal or renal clearance, or is incorporated onto bone surfaces. In the case of ^223^Ra decay product, biodistribution data show less than expected from radioactive equilibrium, activity of ^211^Pb and ^211^Bi in urine and spleen, and some accumulation of ^211^Pb and ^211^Bi in the kidneys. This indicates incomplete retention of decay product inside the liposome. In another report, the same group investigated the distribution and tumour-targeting properties of ^223^Ra encapsulated in the same liposome in a human osteosarcoma xenograft mice model and in a dog model with spontaneous osteosarcoma [58]. In the xenograft model they found higher retention activity in the tumour in comparison to soft tissue. In the dog, the uptake was considerably higher in both calcified and non-calcified tumour metastases of different organs compared to normal tissue. Unfortunately, these promising studies did not continue after 2006.

Further studies focused on the possibility of using inorganic nanocarriers to bind ^223^Ra on the surface of nanoparticles or by incorporating into the nanoparticle structures. Few studies have considered the high similarity between Ba^2+^ and Ra^2+^ cations when incorporating ^223^Ra into the crystal structure of insoluble barium salts. The ionic radii of both cations are nearly identical, 142 pm and 148 pm, respectively [106]. Therefore, it is easy to exchange the Ba^2+^ for Ra^2+^ cations. Simple co-precipitation of Ra^2+^ with BaSO_4_ is widely used in analytical procedures for environmental determination of ^226^Ra. Because BaSO_4_ is not toxic and is easily synthesised for the use of small nanoparticles, Reissig et al. [59] proposed BaSO_4_ nanoparticles as carriers to stably bind radium radionuclides to a targeting molecule. In a one-pot synthesis, radium-doped alendronate-functionalised BaSO_4_ nanoparticles were obtained from [^224^Ra]Ra(NO_3_)_2_, (NH_4_)_2_SO_4_, BaCl_2_ and alendronate. Alendronate, a bisphosphonate containing an amine group, was used as a linker to attach phosphonate groups to the surface of BaSO_4_. The amino group can be used to form a peptide bond with the targeting biomolecule. In optimal conditions, the authors obtained [^224^Ra]BaSO_4_ nanoparticles with a hydrodynamic radius of 140 nm. Unfortunately, it was found that only approximately 20% of ^224^Ra were incorporated into the nanoparticles, whereas 80% of the activity remained in the supernatant solution. The radiolabelled product showed very high stability with <5% activity released. In the next publication, the same group synthesised much smaller ^224^Ra-labelled BaSO_4_ nanoparticles [60]. Moreover, labelling efficiency was better and was ~30%. This was sufficient for future therapeutic applications. In the next step, the authors plan conducting in vitro and in vivo studies to examine the diagnostic and therapeutic properties of targeted bioconjugated [^224^Ra]BaSO_4_.

In another work, Gawęda et al. [69] proposed incorporating ^223^Ra into barium ferrite nanoparticles. Nanoparticles with a diameter of 15–30 nm are obtained in a one-pot hydrothermal synthesis (210 °C, 6.5 h). The labelling efficiency exceeded 90% due to the high similarity of Ra^2+^ and Ba^2+^. No leakage of ^223^Ra was observed in the human serum stability test. Leakage of ^211^Pb (decay product of ^223^Ra) was ~15%. A ethylphosphonoacetate linker was used to attach the targeting biomolecule (Trastuzumab).

In a series of publications, the possibility of using hydroxyapatite (HAP) nanoparticles to immobilise ^223^Ra is described [61,62,63,64]. HAP is a naturally occurring mineral form of calcium phosphate with the formula Ca_5_(PO_4_)_3_(OH). HAP is the main inorganic constituent of bones and teeth and is well tolerated by the living organism. In nuclear medicine, HAP microparticles are used to transport ^177^Lu for liver cancer therapy [107] and can be labelled with ^99m^Tc for the diagnosis of bone cancer [108]. Kozempel et al. [63] tested HAP nanoparticles as a carrier of ^223^Ra. They proposed two strategies for ^223^Ra-HAP nanoparticles preparation: The first method was based on the surface sorption of ^223^Ra on ready-made HAP nanoparticles. The latter was based on the intrinsic incorporation of ^223^Ra into HAP structures during synthesis. In both methods, labelling yields were high and stability was acceptable, though the internal strategy gave slightly better stability results. Surprisingly, no significant release of activity was detected in the stability tests probably due to readsorption of liberated ^211^Bi and ^211^Pb. A similar situation was observed in other nanomaterials like nanozeolites [65] and barium ferrites [69] where readsorption was also the source of large retention of ^223^Ra decay products, ^211^Bi, and ^211^Pb. In subsequent studies devoted to labelling HAP nanoparticles with ^223^Ra, Vasiliev et al. obtained similar results [62]. They found that the optimal labelling of HAP nanoparticles is obtained by the intrinsic incorporation of ^223^Ra into HAP structure during synthesis at pH 4–7, followed by annealing at 900 °C. When synthesised under these conditions, ^223^Ra-HAP nanoparticles exhibited high stability while retaining 95% Ra activity. The same group presented the dynamics of ^223^Ra penetration into porous HAP granules and its redistribution in sorption–desorption processes.

Suchankova et al. [64] tested the labelling of two HAP nanoparticles with ^223^Ra in a Britton–Robinson buffer solution within a pH range of 2 to 12. Both nanomaterials >pH 6 showed a sorption higher than 95% of ^223^Ra. Using the applied chemical equilibrium model, they postulated the most important species playing a role in sorption were RaCO_3_, RaPO_4_, RaHPO_4_ and Ra(Ac)_2_ on the edge sites, and Ra^2+^ and RaH_2_PO_4_^+^ on layer sites.

A promising approach was proposed by Piotrowska et al., using nanozeolites NaA as ^223^Ra carriers [65,66]. Unlike other proposed solutions, ^223^Ra labelling occurs by a Na^+^ for Ra^2+^ ion exchange process after synthesis of the bioconjugate. In other systems, radioactive nanoparticles are first prepared and then modified, which causes necessity of working with radioactive materials and loss of radioactivity by ^223^Ra decay. It was shown that NaA nanozeolite strongly binds radium radionuclides and its decay products [65]. NaA nanozeolites were conjugated by silan linker to substance P(5-11)-peptide exhibited high affinity to NK1 receptor on glioma cell [66]. The ^223^RaA–silane–PEG–SP(5–11) bioconjugate successfully retained 99% of ^223^Ra and 95% of the daughter radionuclides without compromising the tumouricidal radiation properties. This retention was higher than expected for the size of the nanozeolites and was explained by resorption of decay products ^219^Rn and ^211^Pb on the nanoparticle due to high affinity of zeolite for Rn and Pb^2+^ [66]. However, according to Holzwart et al. [37], these results were obtained using nanozeolite labelled with ^223^Ra equilibrated with human serum and cannot be transferred to in vivo models. Blood flow may rapidly dislocate the decay products from the surface of the nanozeolite particles and reduce the resorption probability. However, considering the internalisation rate of the bioconjugate inside the cell, perhaps after reaching the target cells the resorption process will play an important role in preventing of the release free ^211^Pb and ^211^Bi from the cells. Nanozeolite radiobioconjugates have high receptor affinity towards the NK-1 receptor expressing glioma cells in vitro, and exhibits properties suitable for the treatment of glioma cancer cells by intratumoural or post-resection injection [65,66]. Intravenous injection of the ^223^RaA–silane–PEG–SP(5–11) radiobioconjugate for glioma treatment is excluded due to its relatively large size and high hydrophilicity preventing it from crossing the blood–brain barrier.

After successful attempts to immobilise ^225^Ac inside LnPO_4_ nanoparticles [82,83,84,85], a team from the University of Missouri explored using LnPO_4_ nanoparticles to encapsulate two radium radionuclides: ^223^Ra and ^225^Ra [67]. Ac^3+^ can co-crystalise with lanthanum phosphate to form one phase of (Ac)LnPO_4_. However, Ra^2+^ cations most likely form a phosphate mixture, and we observe a weaker binding of ^223,225^Ra compared to ^225^Ac. The ^223^Ra-labelled LnPO_4_ retained ~88% of the ^223^Ra activity over a period of 35 days. For reference, (^225^Ac)LnPO_4_ retained >99% over 21 days [109]. Covering of (^223^Ra)LnPO_4_ core by two cold LaPO_4_ shells reduced the release of ^223^Ra and its daughter, ^211^Pb, to 0.1% over 27 days.

Carbon nanostructures are also being studied as carriers of ^223^Ra. In 2020, Kazakov et al. [70] tested various materials as carriers for selected radionuclides, including ^226^Ra radionuclide, as surrogate of ^223^Ra. They reported that Ra^2+^ sorption occurred only on reduced graphite oxide samples, and reaching 60% activity. However, in PBS and PBS + BSA buffers the desorption went from 35 to 70% in 30 min. Recently, the same team presented the results of ^223^Ra sorption on nanodiamond modified with derivatives of amino acids and EDTA [71]. Unfortunately, the maximum labelling was only 10% at the nanodiamond concentration 380 µg mL^−1^.

### 3.2. Actinum-225

The half-life of 9.92 d and decay energy of ~28 MeV (emission of 4 α-particles) makes the ^225^Ac radionuclide an attractive isotope in targeted α therapy. The half-life of ^225^Ac allows for long-distance distribution, as well as comfortable labelling and administration. Another important advantage of ^225^Ac in nuclear medicine is the emission of 440 keV γ-ray after the decay of the daughter radionuclide—^213^Bi. This can be used for imaging to determine the biodistribution of the radiopharmaceutical in the body. However, similar to ^223^Ra, the release of α-particle-emitting decay products from ^225^Ac-chelate complexes with high recoil energy can cause severe toxic effects in healthy organs and tissues. The use of nanoparticles seems to be very promising therapeutic strategy to entrap potentially radiotoxic daughter nuclides at the tumour site.

Sofou et al. developed pegylated liposomes with different membrane charges (zwitterionic and cationic) and sizes to encapsulate ^225^Ac and daughter nuclides [72]. Zwitterionic liposomes retained more than 88% of ^225^Ac for over 30 days, whereas cationic liposomes only retained 54%. Furthermore, the liposome size is critical in determining daughter nuclide retention. Based on theoretical calculations, the authors suggested that large-sized liposomes, ~650 nm, are required to yield >50% retention of ^213^Bi. However, these predictions did not consider the experiments where ^225^Ac localisation at the phospholipid membrane reduced the ^213^Bi (last daughter) retention up to 7% after 10 days.

To overcome this problem, new complex nanoconstructs were synthesised such as multivesicular liposomes (MUVELs), which are larger liposomes that contain small lipid vesicles [75]. These MUVELs were conjugated to the monoclonal anti-HER2/neu antibody, Trastuzumab, and evaluated in vitro for targeted delivery of ^225^Ac to ovarian cancer cells. The results showed better retention of ^213^Bi (17–18% after 20 days) generated by ^225^Ac in MUVELs compared to the aforementioned liposome strategy [72]. Their biological studies demonstrated higher binding of radiolabelled immunoliposomes to ovarian cancer cells than nontargeted liposomes, but lower immunoreactivity than the radiolabelled antibody. Moreover, the cell uptake kinetics was slower for immunolabelled MUVELs in comparison to free antibody due to different diffusibilities of each structure. Despite some improvements, the maximum entrapment efficiency of ^225^Ac in multivesicular liposomes did not exceed 10% of the total initial activity leading to low specific activities of produced radioactive nanoparticles.

In order to increase the encapsulated radioactivity of ^225^Ac, the same authors applied higher temperature (65 °C) to label the liposome (120 nm nanoconstructs containing 1,2-dinonadecanoyl-sn-glycero-3-phosphocholine lipids and cholesterol) and entrapped DOTA [73]. At this temperature, they observed significant membrane transport of the radionuclide/ionophore complexes and chelation of ^225^Ac by the encapsulated DOTA occurred with minimal thermal hydrolysis of the lipids comprising the membrane. In addition, they tested the maximised radionuclide loading and retention of radioactivity in two types of ionophores (oxine and calcium ionophore A23187) and buffers (citrate and acetate) that play a significant role in enabling cations through lipid membranes. This study showed identical ^225^Ac loading efficacy (~55–73%) of PEGylated liposomes for both ionophores in acetate buffer. By contrast, the radioactivity loading in liposomes was extremely low due to slow kinetics of complex formation between ^225^Ac and the encapsulated DOTA in the presence of citrate buffer. The main fraction release of the entrapped radioactive contents in PBS and 10% serum supplemented media at 37 °C occurred after the first two hours and was stably retained for 30 d. The highest retention of ^225^Ac was observed in PBS (~81 ± 7% of the initially encapsulated radioactivity). The conjugation of Trastuzumab allowed specifically targeting liposomes to SKOV-3-NMP2 cells expressing HER2 receptors. Unfortunately, these immunoliposomes exhibited lower binding capacity in comparison to radiolabelled Trastuzumab.

**Figure 3:**
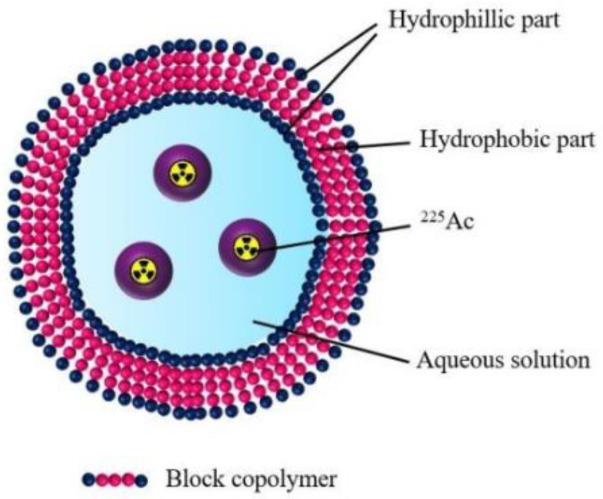
Structure of the polymersomes labelled with ^225^Ac.

The next article of liposome encapsulation of ^225^Ac was published by Bandekar et al. in 2014 [8]. The authors developed ^225^Ac-labelled liposomes for targeted antivascular radiotherapy of prostate cancer. High radioactive ^225^Ac liposomes were conjugated to the J591 monoclonal antibody and the A10 aptamers that recognise the extracellular domain of prostate-specific membrane antigen (PSMA) protein. The loading efficiency ranged from 58% to 86%, which was dependent on the terminated PEG chains. In vitro biological studies showed that radioactive J591-liposomes and ^225^Ac labelled J591antibody exhibit similar cytotoxicity. These radiobioconjugates were more cytotoxic than A10 aptamer-labelled liposomes. Both J591- and A10-labelled liposomes were internalised similarly, ranging between 25 and 36%. These studies demonstrated the dominance of anti-PSMA liposomes loaded with ^225^Ac over both A10-labelled liposomes and nontargeted nanoparticles, signifying the potential of this radiobioconjugate in antivascular α-radiotherapy. The usefulness of liposomes as carriers was also studied by Henriksen et al. [16], where 120 nm liposomes that were loaded with ^228^Ac showed good labelling yield (~61%) and 95% stability in serum after 24 h. Polymersomes are spherical vesicles consisted of polymers and have been proposed for ^225^Ac encapsulation (Figure 3). These nanocarriers are similar to liposomes (Figure 2). However, they consist of amphiphilic block copolymers whilst liposomes are composed of lipid layers. The membrane thickness of polymersomes can vary between 5 and 50 nm, whereas liposomes are thinner (3–4 nm) [109]. In addition, polymersomes exhibit greater stability and less permeability, suggesting they may prevent or at least reduce the release of the recoiling daughter nuclides [79].

The first attempt using polymersomes to encapsulate ^225^Ac focused on the theoretical retention recoiling atoms using Monte Carlo simulations. The results showed that the size and number of block copolymer layers play an important role in the retention of recoiling atoms. Double-layered polymersomes with 400 nm retained 47% of ^213^Bi recoils, whereas retention of single layer polymersomes retained 10–20% lower. Increasing the nanovesicle diameter to 800 nm completely retained ^211^Fr and 80% of the third radionuclide daughter ^213^Bi. Based on Monte Carlo calculations, larger double-layered polymersomes are the most effective in ^225^Ac encapsulation and the retention of ^211^Fr and ^213^Bi recoil daughter nuclides. Furthermore, theoretical computer simulations were verified experimentally [110]. The encapsulation of ^225^Ac radionuclide into polymersomes and its transport through the hydrophobic bilayer into the aqueous cavity was achieved by applying calcium ionophore (A23187) and tropolone. The maximum encapsulation efficiency of ^225^Ac in combination with calcium ionophore capped at 68% and was similar to the previous results presented for lyposomes using the same method [16]. The percentage of retention for ^211^Fr and ^213^Bi in 800 nm vesicles was 69.7 ± 1.5% and 53.7 ± 4%, respectively. These vesicles exhibited the same tendency. However, the retention for ^211^Fr was lower in comparison to theoretical simulations [79]. In the biological studies of 100 nm polymersomes, the internalisation in HeLa cell lines showed that these nanocarriers highly accumulate around the cell’s nucleus, suggesting endocytosis as an actively transporting these molecules that may contribute to the reduction of escaping daughter nuclides.

To improve the recoil retention, ^225^Ac was coprecipitated with InPO_4_ to form small metal-phosphate nanoparticles inside polymersomes [77]. The loading efficiency of ^225^Ac in these nanoparticles was 90%. The results showed 20% higher retention of the first daughter nuclide. Improvement with ^211^Fr was seen in all size vesicles, whereas ^213^Bi retention was improved by 10% in smaller polymersomer sizes in comparison to previous studies with ^225^Ac-DTPA-containing polymersomes. The 100 nm nanocarriers exhibited better retention of both α-emitting daughter nuclides (~20% higher) in comparison to polymersomes without indium phosphate carrier [110]. Next, the therapeutic potential of ^225^Ac incorporated polymersomes in U87 spheroids mimicking tumour was determined [76]. The distribution of radioactive nanocarriers in 3D cell culture was not rapid. It took 4 days to spread throughout the spheroid, whereas after 7 days the distribution of polymersomes was scarcely homogenous. Distribution of nanocarriers in the tumour not only depends on the size of administered vesicles, but also on the shape, surface charge, solubility as well as the type of the cancer [111]. The cytotoxicity studies performed on 3D cell culture using 1 kBq of ^225^Ac-labelled polymersomes showed significant reduction in spheroid size. Interestingly, only 0.1 kBq of activity was sufficient to inhibit spheroid growth, indicating that very low activity of ^225^Ac can inhibit tumour growth. These results demonstrated the high potential of ^225^Ac vesicles in future therapy of glioblastoma. Another publication compared the recoil retention of ^225^Ac DTPA complexes and ^225^Ac coprecipitated with InPO_4_ immobilised in 100 nm polymersomes [78]. In in vivo experiments, synthesised ^225^Ac-nanocarriers were injected intravenously through the tail vein in healthy mice and intratumourally in xenografted mice bearing MDA-MB-231 well-vascularised tumours. After 4 h of the intravenous injection, the recoil retention studies showed that more ^213^Bi was retained in the blood and kidney for metal-phosphate nanoconstructs containing polymersomes than for DTPA nanoparticles, showing the potency of nanocarriers with InPO_4_. Spleen retention was similar for both nanostructures. High ^213^Bi activity was found in the tumour tissue where ^225^Ac-containing DTPA polymersomes were injected intratumourally. ^213^Bi was also completely retained in the tumour when entrapped in 200 nm vesicles. Biodistribution studies in the mouse models showed high uptake of polymersomes in the cancer tissue, whereas ^225^Ac-DOTA complex (used as a control) was rapidly excreted through the kidneys. Additionally, ^225^Ac-polymersomes and ^225^Ac-DOTA significantly inhibited tumour growth and caused γ-H2AX foci (double-stranded breaks), indicating the effectiveness of this α radionuclide therapy. Nevertheless, before ^225^Ac-polymersomes can be used for metastatic cancer therapy, additional studies are needed to find the appropriate size of nanocarriers, circulation time in the blood, retention of daughter nuclides at the therapy site and tumour uptake.

An interesting paper published by Sempkowski et al. [90] investigated sticky lipid nanoparticles loaded with ^225^Ac radionuclide. These vesicles clustered with HER2-targeting peptides on their surface exhibited high reactivity to breast cancer cells with a low expression of receptors like MDA-MB-231 or MCF7 compared to uniformly functionalised nanoparticles (nanoparticles with PEG and HER2 targeting monoclonal antibody). Furthermore, sticky ^225^Ac vesicles caused the death of 42–61% of MDA-MB-231 and MCF7 cancer cells at the extracellular pH of 6.5. Uniform functionalised nanoparticles did not affect cell viability. Upon entering the cell, the sticky vesicles localise fast to the perinuclear area, improving the toxic efficacy of ^225^Ac and the retention of recoiled atoms. These data show that nanoparticles could be a potent therapy for breast cancer patients with low HER2 receptor density, particularly because cytotoxicity for normal breast cells was not observed.

Looking for other nanocarriers for ^225^Ac immobilisation, Mwakisege et al. applied carbon-based nanostructures, fullerenes, to encapsulate ^225^Ac radionuclides because of their chemical and thermodynamic stability [80]. ^225^Ac was entrapped in fullerenes by direct current (DC) arc discharge-catcher method in a He atmosphere. After coupling the fullerene surface with organic adducts, only ~45% of original activity was retained in the cage, and unfortunately, ^221^Fr leakage was also observed. Next, the synthesis of ^225^Ac metallofullerenes and its electronic properties were studied by radiochromatography [81]. ^225^Ac@C_82_ (85 π electrons) species were suggested as the best potential candidates as determined by high-performance liquid chromatography (HPLC). The authors did not perform any retention studies.

The first use of carbon nanotubes as carriers for ^225^Ac was proposed by Ruggiero et al. [88]. Single-wall carbon nanotubes (SWCNT) appended with amines were conjugated to a DOTA ligand and a neovascular-targeting antibody E4G10 that has an affinity for the monomeric vascular endothelial-cadherin (VE-cad) epitope presented in tumour angiogenic vessels. In vivo studies using mice bearing LS174T (colon) adenocarcinomas were treated with radiolabelled high specific activity (SA) SWCNT constructs (851 GBq/g of nanoparticles) and showed two times higher survival and significant tumour regression in comparison to control groups (treated with saline and low SA analogue—1.9 GBq/g) These results demonstrated high specificity of the targeting E4G10 antibody when in combination with α particles and the unique properties of SWCNT, such as rapid renal excretion, enzymatic degradation and nontoxicity. Further studies presented a two-step approach to target tumours with the use of carbon nanotubes [87]. The external sidewall of SWCNTs (350 nm in length and with a diameter of ~1.2 nm) with primary amines was modified with the bifunctional chelator DOTA, attached to a morpholino oligonucleotide complementary to a functionalised antibody (cMORF), and then finally labelled with ^225^Ac. The in vitro results of studies showed that SWNT-cMORF self-assembled onto cancer cells with high specificity. The in vivo mice experiments revealed that multistep therapy, with the use of mAb-MORF followed by SWNT-cMORF-(^225^Ac)DOTA, was very effective and caused complete elimination of the lymphoma tumour. In addition, the rapid clearance of SWNT-cMORF-(^225^Ac)DOTA construct significantly reduced the toxicity five- to tenfold in mice compared to labelled mAb, free ^225^Ac and mAb labelled with MORF. The two-step targeted alpha therapy was found to be feasible and effective.

The application of carbon nanotubes in ^225^Ac^3+^ encapsulation was demonstrated by Matson et al. [89]. These studies were performed with the use of modified SWNT (sidewall defects) known as ultrashort tubes (US-tubes). These nanoparticles were loaded with ^225^Ac^3+^. Three different loading methods were examined: ^225^Ac alone, ^225^Ac^3+^ loaded with Gd^3+^ ions and ^225^Ac^3+^ loaded after the Gd^3+^ ions. The results demonstrated that after ^225^Ac^3+^ loading alone, 95% of the initial activity remained in US-tubes. Simultaneous and sequential adding of ions caused 50% encapsulation of α radionuclide due to the competition of large excess of Gd^3+^. Challenge experiments in human serum showed that only 40% of single-loaded ^225^Ac^3+^ was present inside the nanoparticles. Simultaneous and sequential techniques retained 77% and 80% of this radionuclide, respectively. Unfortunately, for all these ^225^Ac^3+^ studies, retention of ^225^Ac decay products was not determined. These authors provided a novel method to encapsulate two ions which can be used in imaging by MR and α therapy. However, further studies are needed to optimise the process and modify the nanotubes as carriers for targeted treatment.

The synthesis of ^225^Ac-labelled lanthanum phosphate nanoparticles was the next interesting idea to encapsulate ^225^Ac and retain the recoil atoms [84]. Due to the chemical similarity between Ac^3+^ and La^3+^ cations, inorganic nanoparticles with a diameter of 3–5 nm were successfully doped with ^225^Ac (radiochemical yield of ~47%), forming La(^225^Ac)PO_4_. The results showed that ~50% of ^221^Fr and ^213^Bi radionuclides released from the nanoparticles within 6 d and radioactivity remained constant for over 30 d. In the next step, nanoparticles were functionalised with α-hydroxy acid and conjugated to the monoclonal antibody, 201B, targeting thrombomodulin receptors highly expressed in the lung endothelium. Biodistribution and micro SPECT/CT imaging performed on female BALB/c mice revealed rapid and specific uptake of La-(^225^Ac)PO_4_ NPs-mAb in the lungs after intravenous injection. The same in vivo experiments demonstrated that after 1 h post-injection, ~50% of the recoil daughter nuclides were retained at the target site. In contrast to in vitro studies, where 50% of ^213^Bi remained constant for many days, the loss of ^213^Bi decreased significantly down to 10% within 5 d after binding to mouse lung tissue. The likely reason for such low retention of ^213^Bi in the mouse body is because of the entrapment of radionuclide by endothelial cells lining the lung capillaries. This causes less diffusion through the tissue. As was expected, non-targeted lanthanum phosphate nanoparticles accumulated mostly in the liver and spleen due to the recognition by the reticuloendothelial (RE) system. Even the results seem to be significantly improved in comparison to conventional approaches based on the DOTA chelator, still ~50% of recoil atoms present a risk in future therapy with ^225^Ac.

**Figure 4:**
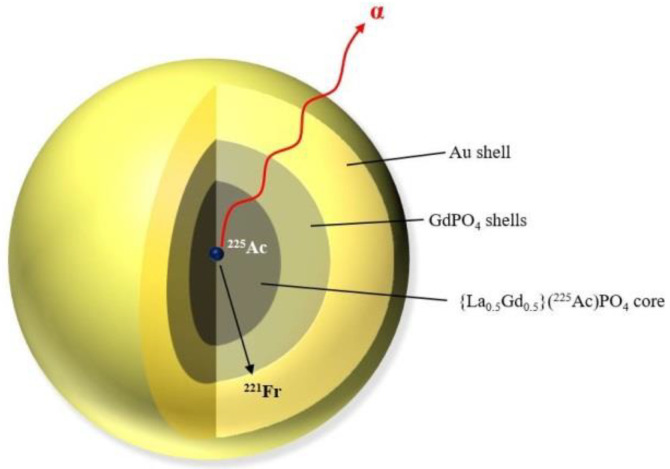
Gold-coated lanthanide gadolinium phosphate nanoparticle (La_0_._5_Gd_0_._5_PO_4_@4 shells GdPO_4_@Au) [84].

A new approach using gold-coated lanthanide phosphate nanoparticles was proposed to reduce the toxicity of escaped daughter nuclides [85]. ^225^Ac was loaded in the {La_0.5_Gd_0.5_}PO_4_ core. A GdPO_4_ layer was used to improve the daughter’s nuclide retention (Figure 4). The Au shell facilitated mAb 201b antibody conjugation reduced the possible Gd toxicity for future in vivo applications. Two layers of GdPO_4_ significantly increased the retention of ^221^Fr from 50% [84] to 70%. Adding four shells retained 98% of ^221^Fr decay daughter, which decreased over one week and stabilised at 88%. Moreover, the multilayered particles retained 99.99% of the ^225^Ac parent radionuclide, over a 3 week period. The composition and type of core also influenced the retention ability. Increased retention of ^225^Ac is higher in nanoparticles in which part of the core contained La [83]. Biodistribution studies showed high binding affinity of {La_0.5_Gd_0.5_}(^225^Ac)PO_4_@GdPO_4_@Au-mAb-201b NPs to the lungs, and were in agreement with SPECT/CT data. Retention of ^213^Bi in the nanoparticles was ~69% in lung tissue after 1 h post-injection, decreasing to 84% after 24 h. A similar trend observed for the spleen and liver. It is worth noting that only 2.8% and 1.5% of the ^213^Bi from the injected dose was observed in the kidneys after 1 and 24 h, respectively.

To minimise the effect derived from phagocytic cells as well as to improve lung-specific uptake, clodronate liposomes were injected into mice 24 h before the injection of mAb-201b conjugated nanoparticles [82]. The results showed ~50% greater accumulation of nanoparticles in the lung compared to mice that were not pretreated with clodronate. Additionally, the retention of ^213^Bi daughters in the lung decreased with time from 70% for 1 h to 91% for 24 h, which was higher than presented previously [83]. In vivo experiments performed on EMT-6 tumour cells demonstrated high cytotoxicity for nanoparticles linked to mAb-201b, leading to significant decrease in lung colonies compared to untargeted nanoparticles. ^225^Ac multilayered nanoconstructs seem to be the most improved technology, and they are very promising for the future alpha therapy.

TiO_2_ nanoparticles used as carriers for ^225^Ac were proposed by Cędrowska et al. [86]. The nanoparticles were labelled with ^225^Ac with a high yield of 99.8% and functionalised with silane-PEG-SP(5-11) conjugates (average 80 molecules per one nanoparticle) to target NK1 receptors overexpressed in gliomas. ^225^Ac^3+^ cations were adsorbed on the surface of TiO_2_ nanoparticles through an ion-exchange reaction with hydroxyl groups. Retention of ^225^Ac and ^221^Fr in PBS or NaCl solutions after 4 days ranged from 95 to 98%. In cerebrospinal fluid (CSF), the retention decreased to ~70%. In glioblastoma cancer cells, the cytotoxicity of ^225^Ac-TiO_2_-silane -PEG-SP(5-11) radiobioconjugate was higher in comparison to non-targeted or non-radioactive nanoparticles, showing the potential of nanoconstructs in TAT of brain tumours.

**Figure 5:**
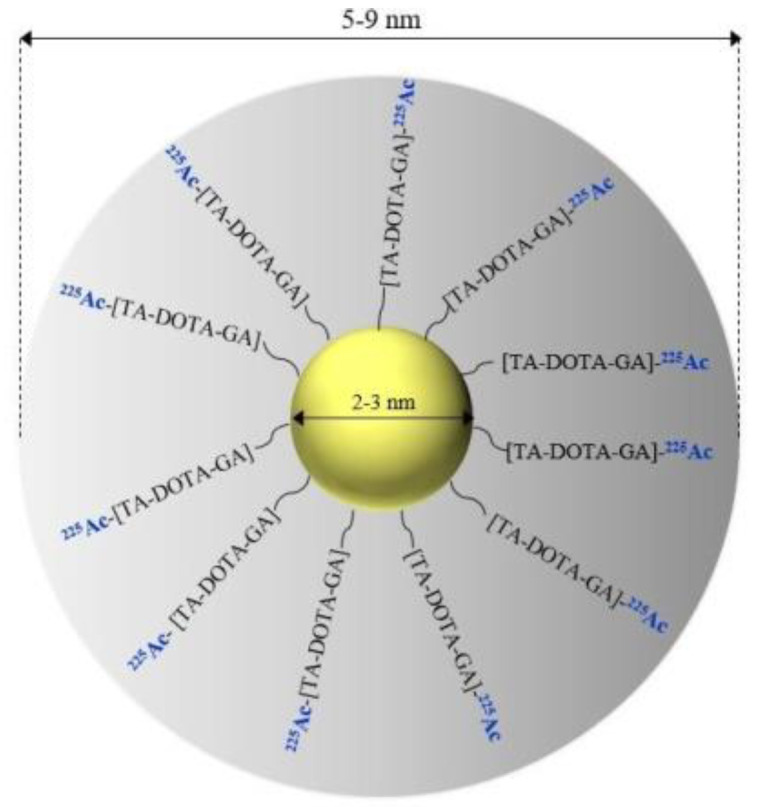
Schematic of an ^225^Ac-Au@TADOTAGA radiobioconjugate.

In another work proposed by Salvanou et al., ^225^Ac radionuclide was conjugated to 2–3 nm gold nanoparticles via a DOTA-derivative chelator (TADOTAGA) for local radiation treatment of cancer (Figure 5) [52]. Radiochemical yield assessed by ITLC was ~86%. In vitro experiments performed on U87 glioblastoma cancer cells showed significant cytotoxicity of ^225^Ac-Au@TADOTAGA radioconjugate. After 48 h of treatment with 0.5 kBq/mL synthesised nanoparticles, less than 30% of cells were detected as viable. Intravenous injection of ^225^Ac-Au@TADOTAGA in U87 MG tumour-bearing SCID mice caused predominate accumulation in the kidneys, liver and spleen. Biodistribution studies after intratumoural injection showed high tumour uptake at 2 h post-injection (60.67 ± 3.87% IA/g), which slowly decreased over time. The preliminary therapeutic efficacy studies performed over a period of 22 days revealed tumour growth retardation upon intratumoural injection of ^225^Ac-Au@TADOTAGA in comparison to mice injected with normal saline. These results are very promising. However, further preclinical evaluations are needed to use this radioconjugate as an injectable radiopharmaceutical for local radiation treatment of tumour.

Gadolinium vanadate nanoparticles have been proposed as a carrier for two α-emitters: ^225^Ac and ^227^Th. Gonzalez et al. explored multifunctional gadolinium vanadate core–shell nanoparticles doped with europium ions and ^225^Ac radionuclide [92]. Gd_0.8_Eu_0.2_VO_4_ core–shell nanoparticles were successfully obtained by precipitating Ln^3+^ and ${\mathrm{VO}}_{4}^{3-}$ ions, using sodium citrate as a complexing agent. ^225^Ac and decay products retention was assessed between Gd_0.8_Eu_0.2_VO_4_ core and core +2 nonradioactive shells. The radiochemical yield for ^225^Ac in Gd_0.8_Eu_0.2_VO_4_ core was 58.3%, whereas the additional two Gd_0.8_Eu_0.2_VO_4_ nonradioactive shells on the nanoparticle core increased the yield to 94.8%. ^211^Fr leakage reached a maximum of ~67.6% after 28 days in dialysis, which was higher compared to nanoparticles with two added shells (45.5%) and lanthanum phosphate nanoconstructs reported previously [83,84,85]. Due to the presence of the citrate groups on the nanoparticle surface, the retention of ^213^Bi was greater than ^211^Fr, reaching <15% and ~22% leakage from core and core +2 shells, respectively. Considering the intrinsic properties like luminescence and magnetic functionalities, the radionuclide retention capabilities, the small particle size (below 10 nm) and short time required for the synthesis (<1 h), Gd_0.8_Eu_0.2_VO_4_ core nanoparticles show great potential for medical applications, including α therapy.

The same group proposed gadolinium vanadate nanocrystals (NCs) as carriers of ^225^Ac and ^227^Th radionuclides and contrast agents [91]. The synthesis of GdVO_4_ core and core +2 shell NCs with a tetragonal structure was performed using the same method as previously described [92]. The maximum leakage of ^225^Ac from the NCs core was ~15% and decreased to 2.4% with the addition of two nonradioactive shells. The leakage of ^221^Fr from Gd(^225^Ac)VO_4_ after 23 days in dialysis reached 69.5%, and was improved to 20% by adding two shells. These retention results for ^221^Fr in Gd(^225^Ac)VO_4_ are comparable to those using 100 nm polymersomes, which retained 37% of this radionuclide [110], whereas the retention of ^225^Ac and ^221^Fr radionuclides in Gd(^225^Ac)VO_4_/2GdVO_4_ NCs are close to La_0.5_Gd_0.5_PO_4_ core + 2 shell nanoparticles [83]. Similarly to previous studies, the leakage of ^213^Bi from Gd(^225^Ac)VO_4_ and Gd(^225^Ac)VO_4_/2GdVO_4_ was 22.5% and 19.6%, respectively [92].

### 3.3. Astatine-211

As mentioned before, the carbon–astatine bond is considerably weaker than the carbon–iodine bond and many ^211^At-labelled vectors, which exhibit excellent ex vivo stability dehalogenate in vivo [93]. Because of these problems, others solutions for ^211^At labelling, including nanotechnology, have been reported. Hartman et al. tested the use of 20–50 nm US-tubes as a carrier for ^211^At in targeted α radionuclide therapy [93]. The idea was to introduce ^211^At inside the nanotube. They found that a more stable conjugate is formed when ^211^At is present as the mixed halogen (^211^AtCl) than in the anionic (^211^At^-^) form. The labelling yield of US-tubes with ^211^AtCl is 91.3%, which is much better than labelling with ^211^At^-^ (24.7%). The authors found that a small quantity of ^211^AtCl remained on the exterior of the US-tube sidewall defect sites or tube ends during synthesis. After a simple wash with metabisulfite, the retention of ^211^AtCl@US-tubes was 60.7%. The overall labelling yield of the ^211^AtCl@US-tubes was comparable to the other elaborated astatination methods [48,49,50,51,52]. An important feature of functionalised SWNTs as drug carriers is their rapid clearance via renal glomerular filtration, despite their large molecular weights [112]. This clearance is called fibrillar pharmacology [113] and contrasts with large protein pharmacokinetic profiles.

Kućka et al. [94] and Cędrowska et al. [95] used astatine’s affinity towards metallic silver and proposed the application of silver nanoparticles and silver impregnated TiO_2_ labelled with ^211^At for cancer therapy. The surface of nanoparticles was coated with a hydrophilic polymer, poly(ethylene oxide). In both papers, the effect of the labelling yield using different reducing and oxidising agents was studied. It was found that in reducing conditions, where astatine exists as At^-^, labelling was nearly 100%. Under oxidising conditions, the labelling decreased to about 50% [94]. The Ag-labelled nanoparticles were stable even in a large excess of competing chloride ions [94] and in PBS, cysteine and glutation solutions. In human blood serum, 5% of leakage was observed after 1 h, which increased to 7.8% after 14 h.

In the next three studies, Bilewicz et al. applied the discovered high affinity of astatine to the gold surface for astatination of peptide, substance P (5-11) and monoclonal antibody, Trastuzumab [96,97,114]. Based on DFT calculations of At–Au interactions, and taking in account the proposed reaction between At^−^ and gold clusters:

At^−^ + Au_n_ + H_2_O → Au_n_At + ½H_2_ + OH^−^

the authors found that the source of the gold cluster’s high affinity to At is related to the least negative oxidation–reduction potential of astatine (6 kcal/mol) in the halogen group [114]. In the experimental studies, gold nanoparticles with 5 and 15 nm diameters were modified with substance P(5-11), a peptide fragment which targets the NK1 receptors on the glioma cells [96]. The monoclonal antibody specific for HER2 receptors, Trastuzumab, was also attached [97]. The obtained bioconjugates were quantitatively labelled with ^211^At by chemisorption on the gold surface. The labelled bioconjugates almost retained ^211^At in human serum and cerebrospinal fluid at 37 °C for 24 h. Additionally, in vitro biological studies indicated that ^211^At-Au-PEG-substance P(5-11) radiobioconjugate exhibited a high cytotoxic effect in vitro on glioma cancer cells [96]. The performed studies on HER2-overexpressing human ovarian SKOV-3 cells indicated high internalisation of ^211^At-AuNP-PEG-Trastuzumab in the cell and localisation of radiobioconjugates in the perinuclear area and its high cytotoxicity [97].

### 3.4. Lead-212

^212^Bi (t_1/2_ = 60.5 min) is a potentially interesting α-emitting radionuclide for internal alpha therapy. Unfortunately, its short half-life often limits the application of ^212^Bi to situations when the tumour cells are rapidly accessible to the targeting agent. To expand the range of applications, an interesting method uses the parent radionuclide, ^212^Pb (t_1/2_ = 10.6 h), which generates in vivo ^212^Bi [115]. In comparison to ^212^Bi radiopharmaceuticals, ^212^Pb have a much broader applicability because the half-life of ^212^Pb corresponds better with the pharmacokinetics of various biomolecules. ^212^Pb is transformed to ^212^Bi trough β^-^ decay. Due to small mass of electrons, the calculated recoil energy of the Bi nucleus is ~0.5 eV and is not sufficient to break a chemical bond, which requires ~10 eV. However, over 30% of the γ-rays emitted during ^212^Pb decay are internally converted. The resulting conversion cascade and Auger electrons brings ^212^Bi to highly ionised states, such as Bi^5+^ and Bi^7+^. Therefore, the energy required to neutralise the charge is sufficient to break chemical bonds [116]. Previous attempts to prepare a potential in vivo generator with ^212^Pb complexed by the DOTA chelator failed, because ~36% of Bi escaped as a result of the radioactive decay ^212^Pb→^212^Bi [115]. Because the free radiobismuth escapes the complex during the decay, toxicity emerges when unchelated ^212^Bi accumulates in various organs, mainly in kidneys. Thus, alternative chelators for ^212^Pb complexation need to be further explored. Problems with the retention of the decay product (^212^Bi) is much smaller than α-decayed ^225^Ac or ^223^Ra because the recoil energy after β^-^ decay is ~2 × 10^5^ lower than after α decay.

Henriksen et al. [99] made the first attempts to incorporate ^212^Pb into nanostructures. Containers with incorporated ^212^Pb/^212^Bi were prepared by ionophore-mediated loading of ^212^Pb into liposomes. Uptake of n.c.a. ^212^Pb in liposome was 65% but increased to 90% after addition of a Pb^2+^ carrier. At least 95% of the ^212^Pb and ^212^Bi activity was retained in the liposomes. The performed studies indicate that liposomes give high retention of ^212^Pb and ^212^Bi formed from the ^212^Pb β^-^ decay.

Montafon et al. incorporated ^212^Pb into indium-DTPA-tagged liposomes [98]. They found that the origin of the encapsulation is related to the dynamics of the surface, which makes the membrane partially permeable. Therefore, no ligand was necessary to allow the transfer of ^212^Pb from the external solution to the liposome internal part. A significant improvement of labelling was observed when DTPA is present in the internal part of liposome. Forming a strong complex with Pb^2+^ accelerated the concentration of the ^212^Pb inside the liposome. In optimised conditions, the labelling yield is ~75% and can be obtained with a mean value of 2–3 lead atoms per liposome. Unfortunately, no stability tests have been performed.

An interesting solution using fullerene for ^212^Pb/^212^Bi immobilisation was proposed by Diener et al. [101]. They labelled C_60_ by the recoiled ^212^Pb from α decay of its parent, 0.15 s ^216^Po, generated in situ from the decay of ^224^Ra (t_1/2_ = 3.66 d). Unfortunately, the yield of incorporating^212^Pb into C_60_ was very low, about 0.1 to 0.6%. So to produce one therapeutic dose of ^212^Pb@C_60_ (100 MBq), about 50 GBq of ^224^Ra is needed [8]. Also C_60_ cannot retain inside ^212^Bi formed by β^-^ decay of ^212^Pb.

### 3.5. Thorium-227

^227^Th belongs to the actinium series, and the ^227^Th decay chain contains emission five α-particles to reach stable ^207^Pb (Figure 1).^227^Th emits α particle with energy of 5.9 MeV. However, ^227^Th and all its daughter nuclides deposited at a target tissue produce 34 MeV of energy, the most among the α-emitters studied in cancer therapy. The daughter of ^227^Th is ^223^Ra, which is the first in class α-emitter approved for castration-resistant prostate cancer. The gadolinium vanadate nanoparticles, after tests with ^225^Ac, were proposed as carriers for ^227^Th [92]. The radiochemical yield of ^227^Th encapsulation in core + 2 shell nanoparticles was 81.9%, less than in the case of ^225^Ac. The leakage of immobilised ^227^Th within Gd(^227^Th)VO_4_ reached 1.6% after 12 days and decreased to less than 1.5% in the case of Gd(^227^Th)VO_4_ nanoparticle + two shells GdVO_4_ layers_._ The first decay daughter (^223^Ra) retention was increased after the addition of two nonradioactive GdVO_4_ shells from 61% to 75%. The labelling yield of GdVO_4_ was lower compared to DOTA or HOPO radiobioconjugates, but retention of ^223^Ra was drastically higher [117,118]. The ability of GdVO_4_ core–shell nanoparticles to retain radionuclides gives them the potential to increase specific activity and the possibility to functionalise to make them suitable for targeted therapy because of proton relaxivity for using magnetic resonance imaging.

## 4. Conclusions

Radionuclide delivery systems using nanoparticles have great potential in the field of nuclear medicine. Today, immobilisation of ^223^Ra in inorganic nanoparticles seems to be the only possibility of the use ^223^Ra in targeted alpha therapy. Similarly, recently proposed targeted nanobrachytherapy using nanoparticles labelled with α-emitters has great potential for the treatment of small tumours and tumour metastases. However, an attempt for the immobilisation in nanostructure radionuclides, such as ^225^Ac and ^212^Pb, that can be complexed by “classical” chelators does not seem realistic. Recently, clinical trials for cell internalising radiobioconjugates such as ^225^Ac-PSMA-617 or ^225^Ac-DOTA-octreotide, where ^225^Ac was chelated by DOTA ligand, showed their exceptional therapeutic efficacy, while the toxic effects induced by the release of decay ^213^Bi were negligible. Therefore, there is no need in this case to use nanotechnology to immobilise ^225^Ac and its decay products, as we know nanoparticles after injection, before they reach the tumour, usually accumulate in such critical organs as spleen, liver or lungs causing damage of these organs. This problem remains to be solved.

## Figures and Tables

**Table 1:** Physical properties of α-emitting radionuclides of interest for medical applications.

Radionuclide	Half-Life	Energy of Emitted α Particles (MeV)	Main Production Route	Availability
^212^Bi	60.6 min	6.051 (25.1%) 6.090 (9.8%) 8.785 (64%)	^224^Ra/^212^Bi generator	Relatively high
^213^Bi	45.6 min	5.875 (2.2%) 8.376 (97.8%) ^1^	^225^Ac/^213^Bi generator	Moderate
^225^Ac	10.0 d	5.732 (8.0%) 5.791 (8.6%) 5.793 (18.1%) 5.830 (50.7%)	^229^Th/^225^Ac ^226^Ra(p,2n)^225^Ac ^232^Th(p,spall.) ^225^Ac	Moderate
^211^At	7.2 h	5.870 (41.8%) 7.450 (58.2%)	^nat^Bi(α,2n)^211^At	Moderate
^223^Ra	11.4 d	5.540 (9.0%) 5.607 (25.2%) 5.716 (51.6%) 5.747 (9.0%)	^227^Ac/^223^Ra generator	Commercially available
^224^Ra	3.6 d	5.449 (5.0%) 5.685 (94.9%)	^228^Th/^224^Ra generator	Relatively high
^226^Th	30.7 min	6.234 (22.8%) 6.337 (75.5%)	^230^U/^226^Th generator	Low
^227^Th	18.7 d	5.709 (8.3%) 5.713 (5.0%) 5.757 (20.4%) 5.978 (23.5%) 6.038 (24.2%)	^227^Ac/^227^Th generator	Moderate
^212^Pb	10.6 d	6.051 (25.1%) ^2^ 6.090 (9.8%) ^2^ 8.785 (64%) ^2^	^224^Ra/^212^Pb generator	Commercially available

^1^ energy of α particles emitted by daughter-^213^Po; ^2^ energy of α particles emitted by daughter-^212^Bi.

**Table 2:** Functional nanomaterials labelled with alpha-emitting radionuclides.

Radionuclide	Nanomaterials	Attached Vector	Targeting Cancer	Reason of Using Nanomaterials	Ref.
^223^Ra	Liposome	folic acid and F(ab’)2(IgG1)	Ovarian carcinoma cell line, OvCar-3	Recoil, radionuclide immobilisation	[16]
	Doxorubicin-containing-liposomes (Caelyx^®^/Doxil^®^)	folic acid and F(ab’)2(IgG1)	-	Radionuclide immobilisation	[57]
	Doxorubicin-containing-liposomes (Caelyx^®^/Doxil^®^)	-	-	Radionuclide immobilisation	[58]
	Alendronate functionalized [^223^Ra] barium sulphate	-	-	Radionuclide immobilisation	[59]
	Very small (<10 nm) alendronate functionalized [^223^Ra] barium sulphate	-	-	Radionuclide immobilisation	[60]
	Hydroxyapatite	-	-	Radionuclide immobilisation	[61]
	Hydroxyapatite	-	-	Radionuclide immobilisation	[62]
	Hydroxyapatite	-	-	Radionuclide immobilisation	[63]
	Hydroxyapatite	-	-	Radionuclide immobilisation	[64]
	TiO_2_	-	-	Radionuclide immobilisation	[64]
	Nanozeolite A	-	-	Recoil, radionuclide immobilisation	[65]
	Nanozeolite A	Substance P (5-11)	Glioma cells with NK1 receptor	Recoil, radionuclide immobilisation, nanobrachyterapy	[66]
	Lanthanum phosphate	-	-	Radionuclide immobilisation	[67]
	Superparamagnetic iron oxide nanoparticles	-	-	Radionuclide immobilisation	[68]
	Barium ferrite	Trastuzumab	Breast and ovarian cancer cells with SKOV-3 receptor	Recoil, radionuclide immobilisation	[69]
	Reduced graphite oxide	-	-	Radionuclide immobilisation	[70]
	EDTA functionalized-nanodiamond	-	-	Radionuclide immobilisation	[71]
^225^Ac	Pegylated liposomes	-	-	Recoil	[72]
	Pegylated liposome	PSMA J591 antibody	(PSMA)–expressing cells	Recoil	[8]
	Pegylated liposome	Trastuzumab	SKOV-3 ovarian cells	Recoil	[73]
	pH-tunable liposomes	-	-	Recoil	[74]
	Pegylated multivesicular Liposomes	Trastuzumab	SKOV-3 ovarian cells	Recoil	[75]
	Polymersome	-	-	Recoil	[76]
	InPO_4_ nanoparticles inside polymersomes	-	-	Recoil	[77]
	Polymerosomes	-	-	Recoil	[78]
	Polymerosomes	-	-	Recoil	[79]
	Fullerenes	-	-	Recoil	[80]
	Fullerenes	-	-	Recoil	[81]
	(^225^Ac,Ga_0.5_,La_0.5_) PO_4_@4GdPO_4_@Au	MAb 201b	EMT-6 lung tumour cells	Recoil	[82]
	(^225^Ac,Ga_0.5_La_0.5_) PO_4_@2-4GdPO_4_@Au	-	-	Recoil	[83]
	La (^225^Ac)PO_4_	MAb 201b	EMT-6 lung tumour cells	Recoil	[84]
	(^225^Ac,Ga_0.5_,La_0.5_) PO_4_@4GdPO_4_@Au	MAb 201b	EMT-6 lung tumour cells	Recoil	[85]
	TiO_2_	Substance P (5-11)	NK1 glioma receptor	Recoil	[86]
	Carbon nanotubes-DOTA	LintuzumabRituximab anti-A33	CD20+B-cell lymphoma C33+myelocytic leukaemia A33+ colon adenocarcinoma	Targeted amplified delivery, fast clearance	[87]
	Carbon nanotubes-DOTA	Tumour neovascular-targeting antibody	LS174T xeno-graft tumour model	Targeted amplified delivery, fast clearance	[88]
	Carbon nanotubes	-	-	Increasing specific activity, fast clearance	[89]
	Lipid vehicle	Trastuzumab	BT-474, MDA-MB-231, MCF7 breast carcinoma cell	Targeting cells with low expression of HER 2	[90]
	DOTA gold	-	-	Nanobrachyterapy	[52]
	Gadolinium vanadate	-	-	Recoil, multimodality	[91]
^227^Th	Eu^3+^ doped gadolinium vanadate	-	-	Recoil, multimodality	[92]
^211^At	Ultrashort nanotubes	-	-	Radionuclide immobilisation	[93]
	Silver nanoparticles	-	-	Radionuclide immobilisation	[94]
	Silver impregnated TiO_2_	-	-	Radionuclide immobilisation	[95]
	Gold nanoparticles	Substance P (5-11)	Glioma cells with NK1 receptor	Radionuclide immobilisation	[96]
	Gold nanoparticles	Trastuzumab	Breast and ovarian cancer cells with SKOV-3 receptor	Radionuclide immobilisation	[97]
^212^Pb	Indium tagged liposomes	-	-	Recoil	[98]
	Sterically stabilized liposomes	-	-	Recoil	[99]
	Hydroxyapatite	-	-	Radionuclide immobilisation	[100]
	Water soluble C_60_	-	-	Recoil, Radionuclide immobilisation	[101]
